# Benefits of adversity?! How life history affects the behavioral profile of mice varying in serotonin transporter genotype

**DOI:** 10.3389/fnbeh.2015.00047

**Published:** 2015-03-03

**Authors:** Carina Bodden, S. Helene Richter, Rebecca S. Schreiber, Vanessa Kloke, Joachim Gerß, Rupert Palme, Klaus-Peter Lesch, Lars Lewejohann, Sylvia Kaiser, Norbert Sachser

**Affiliations:** ^1^Department of Behavioural Biology, University of MuensterMuenster, Germany; ^2^Otto Creutzfeldt Center for Cognitive and Behavioral Neuroscience, University of MuensterMuenster, Germany; ^3^Institute of Biostatistics and Clinical Research, University of MuensterMuenster, Germany; ^4^Department of Biomedical Sciences, University of Veterinary MedicineVienna, Austria; ^5^Division of Molecular Psychiatry, Laboratory of Translational Neuroscience, Department of Psychiatry, Psychosomatics, and Psychotherapy, University of WuerzburgWuerzburg, Germany; ^6^Department of Behavioral Biology, University of OsnabrueckGermany

**Keywords:** life history, 5-HTT, anxiety-like behavior, predictive adaptive response hypothesis, match-mismatch

## Abstract

Behavioral profiles are influenced by both positive and negative experiences as well as the genetic disposition. Traditionally, accumulating adversity over lifetime is considered to predict increased anxiety-like behavior (“allostatic load”). The alternative “mismatch hypothesis” suggests increased levels of anxiety if the early environment differs from the later-life environment. Thus, there is a need for a whole-life history approach to gain a deeper understanding of how behavioral profiles are shaped. The aim of this study was to elucidate the effects of life history on the behavioral profile of mice varying in serotonin transporter (5-HTT) genotype, an established mouse model of increased anxiety-like behavior. For this purpose, mice grew up under either adverse or beneficial conditions during early phases of life. In adulthood, they were further subdivided so as to face a situation that either matched or mismatched the condition experienced so far, resulting in four different life histories. Subsequently, mice were tested for their anxiety-like and exploratory behavior. The main results were: (1) Life history profoundly modulated the behavioral profile. Surprisingly, mice that experienced early beneficial and later escapable adverse conditions showed less anxiety-like and more exploratory behavior compared to mice of other life histories. (2) Genotype significantly influenced the behavioral profile, with homozygous 5-HTT knockout mice displaying highest levels of anxiety-like and lowest levels of exploratory behavior. Our findings concerning life history indicate that the absence of adversity does not necessarily cause lower levels of anxiety than accumulating adversity. Rather, some adversity may be beneficial, particularly when following positive events. Altogether, we conclude that for an understanding of behavioral profiles, it is not sufficient to look at experiences during single phases of life, but the whole life history has to be considered.

## Introduction

Individual differences in behavioral traits have been related to differences in reproductive success (e.g., Alcock, 2005), susceptibility to disease (e.g., Henry, 1982; von Holst, 1998; Korte et al., 2005), and the overall quality of life (e.g., Broom, 2006). Accordingly, the comprehensive understanding of the behavioral profile and its shaping through both genetic and environmental factors gains more and more importance in diverse fields, ranging from behavioral ecology to biopsychological and biomedical research (von Holst, 1998; Brown et al., 1999; Gross and Hen, 2004; Korte et al., 2005; Sachser et al., 2013). Concerning the role of the environment, extensive research on animals and humans has shown that both positive and negative experiences play a crucial role in the development of the behavioral profile (e.g., Hennessy et al., 2009; Sachser et al., 2011; van der Doelen et al., 2013). In rodents for example varying quality of maternal care (e.g., Liu et al., 1997; Caldji et al., 1998; Meaney, 2001), complexity of environmental enrichment (Marashi et al., 2003, 2004; Gross et al., 2011), social defeat (Rodgers and Cole, 1993; Jansen et al., 2010; Kloke et al., 2011), and mating experience (Rodríguez-Manzo et al., 1999; Edinger and Frye, 2007) can shape the adult behavior in an either anxiety-reducing or -enhancing way. However, so far most experimental work has focused on single phases of life rather than incorporating a whole-life history approach, which would combine a series of experiences made during different life stages and, hence, would much better represent natural conditions.

The need for such an approach becomes particularly apparent in the context of psychological and biomedical research, where psychiatric illness, such as anxiety disorders, depression, or schizophrenia, does not necessarily develop because of a single adverse event, but rather as a consequence of accumulating experiences during life and/or genetic predisposition, as proposed in the “double-hit hypothesis” (Bayer et al., 1999; Walker et al., 2009) or the “three-hit hypothesis” (Daskalakis et al., 2013). The former assumes that an early-life genetic or environmental insult during a sensitive period of development (“first hit”) establishes a vulnerability to subsequent adversity in later life (“second hit”). Both hits together (“double hit”) are believed to increase the susceptibility to disease (Bayer et al., 1999; Walker et al., 2009). A slightly different view has been postulated in the “three-hit hypothesis” which proceeds on the assumption that the interaction of genetic (“first hit”) and early-life environmental factors (“second hit”) shapes a phenotype that is more or less vulnerable to later-life environmental conditions (“third hit”). The “allostatic load hypothesis” (McEwen and Stellar, 1993; McEwen, 2003) considers the accumulation of adverse experiences over the lifetime to be a major causative risk factor for the development and maintenance of disease (McEwen, 2003). Allostatic load is defined as wear and tear on the body and brain resulting from chronic hyper- or inactivity of physiological systems that are normally involved in adaptation to environmental challenge (McEwen, 2003). However, this concept does not explain why negative health outcomes do not necessarily arise in the aftermath of adverse life events, but can, on the contrary develop under beneficial conditions (Homberg, 2012).

More recently, an alternative view has been proposed and referred to as predictive adaptive response hypothesis, which suggests that cues received in early life influence the development of a phenotype in such a way that it is normally adapted to later environmental conditions (Bateson et al., 2014). In case that the predicted and actual environments differ, adverse consequences for health can arise due to the experienced mismatch between the early programming and later actual environment (Bateson et al., 2004, 2014; Gluckman et al., 2005a,b, 2007; Heiming and Sachser, 2010; Sachser et al., 2011, 2013). Therefore, consistent (“matching”) conditions can promote successful adaptation and health, whereas a discrepancy (“mismatch”) between the early and late environment results in maladaptation and hence may cause disease (Bateson et al., 2004; Gluckman et al., 2005a,b, 2007; Schmidt, 2011). In accordance with this hypothesis, recent studies gained first evidence in rodents that the experience of early adversity can indeed bring about advantages under challenging conditions later in life (Champagne et al., 2008; Oitzl et al., 2010; Oomen et al., 2010).

Actually, the vast majority of research on environmental influences has been performed in rodent models. Most studies focused on the prenatal and early postnatal phase, when brain circuits are shaped and experiences are assumed to exert long-lasting changes in behavior and physiology (Cratty et al., 1995; Seckl, 2004; Champagne and Curley, 2005; Kaiser and Sachser, 2005). Indeed, mild adversity during early stages of life, e.g., exposure to olfactory cues that signal danger (Mandillo and D’Amato, 1997; Heiming et al., 2009, 2011), has been shown to elicit increased levels of anxiety later in life (Vallée et al., 1997; Meaney, 2001). Apart from the perinatal period, too little attention has been paid to another sensitive developmental phase: the adolescence (Spear, 2000; Sachser et al., 2013). During the transition from childhood to adulthood substantial remodeling of the brain occurs in response to hormonal and physical changes. Hence, the brain is particularly sensitive to environmental and social experiences, such as losing or mating experiences (Miczek, 1979; Rodríguez-Manzo et al., 1999; Edinger and Frye, 2007; Schmidt et al., 2007; McCormick et al., 2008; Jansen et al., 2010; Kloke et al., 2011; Lürzel et al., 2011; Raftogianni et al., 2012). Finally, experiences during adulthood have also been demonstrated to profoundly influence the behavior and physiology (Buwalda et al., 2005; Jansen et al., 2010). Despite the increasing knowledge on how single effects impinge on the behavioral profile, it is not yet clear whether and how these effects interact with each other when they are combined in a life history approach.

Apart from environmental influences, genetic predispositions play an important role in modulating the behavioral profile (Caspi et al., 2003, 2010; Canli and Lesch, 2007). Several so-called candidate genes have been identified with respect to the genetic basis of anxiety and anxiety disorders, for instance different variants in genes encoding the serotonin transporter (5-HTT), tryptophan hydroxylase-2, monoamine oxidase A (for a review see Burmeister et al., 2008). In humans, a repeat length polymorphism (5HTTLPR) in the transcriptional control region of the 5-HTT gene (*SLC6A4*) was found, resulting in allelic variation of 5-HTT expression and function, and associated traits of negative emotionality including anxiety and depression (Collier et al., 1996; Lesch et al., 1996; Caspi et al., 2003, 2010; Canli and Lesch, 2007). The generation of mice with a targeted disruption of the 5-HTT gene allows to investigate the consequences of its diminished or absent function. Indeed, 5-HTT knockout mice were shown to display increased anxiety-like behavior, thus providing a valuable animal model to also analyze interactions between 5-HTT gene variation and different life experiences (Holmes et al., 2003a,b; Carroll et al., 2007; Heiming and Sachser, 2010).

Based on the knowledge that behavioral traits are shaped during different stages of life—ranging from the prenatal stage to adulthood—and that social experiences can enhance or attenuate anxiety-like and exploratory behavior, the aim of the present study was to investigate the effects of four different combinations of positive and negative socio-environmental conditions over the course of life on the behavior of male mice, particularly with regard to matching and mismatching conditions. As the 5-HTT genotype is also known for its modulating effects on anxiety-related traits, we focused on the shaping of anxiety-like behavior and exploratory locomotion by different life histories in wildtype (+/+), heterozygous (+/−), and homozygous (−/−) 5-HTT knockout mice. We therefore sought to test the hypotheses that different life histories and/or variations in the 5-HTT genotype lead to differences in anxiety-like behavior and/or exploratory locomotion.

## Materials and methods

### Animals and housing conditions

In the present study, 5-HTT knockout mice (Bengel et al., 1998), backcrossed into a C57BL/6J genetic background for more than 10 generations, were used. All animals originated from the internal stock of the Department of Behavioral Biology, University of Muenster, Germany. The original breeding stock was provided by the Division of Molecular Psychiatry, University of Wuerzburg, Germany. Breeding pairs consisted of a male and female 5-HTT +/− mouse each, resulting in 5-HTT +/+, 5-HTT +/−, and 5-HTT −/− offspring. Genotyping was accomplished by using ear tissue to extract genomic DNA, which was amplified by PCR. DNA fragments of either 225 bp (5-HTT +/+), 272 bp (5-HTT −/−), or both (5-HTT +/−) were identified by agarose gel electrophoresis. All mice were housed in transparent standard Makrolon cages type III (38 cm × 21 cm × 15 cm) with sawdust as bedding material (Allspan, Höveler GmbH and Co.KG, Langenfeld, Germany), a paper towel as nesting material, and food (1314 for breeding females, 1324 for all other mice, Altromin GmbH, Lage, Germany) and water provided *ad libitum*. Additionally, the breeding females were given supplementary oat flakes (Fortin GmbH and Co.KG, Duesseldorf, Germany). Housing rooms were maintained at a 12 h light/dark cycle with lights on at 8:00 am, a temperature of 22 ± 2°C, and a relative air humidity of 50 ± 10%.

In total, 124 adult 5-HTT +/− females were each mated with one of 21 adult 5-HTT +/− males. The male offspring of all three genotypes (*n*_5-HTT+/+_ = 40, *n*_5-HTT+/−_ = 48, *n*_5-HTT−/−_ = 34) were used for the experiment.

All procedures complied with the regulations covering animal experimentation within the EU (European Communities Council DIRECTIVE 2010/63/EU). They were conducted in accordance with the institution’s animal care and use guidelines and approved by the national and local authorities (reference number: 84-02.05.20.12.212). Moreover, all efforts were made to minimize the number of animals used and the intensity of the procedures applied.

### Experimental design

**Figure 1:**
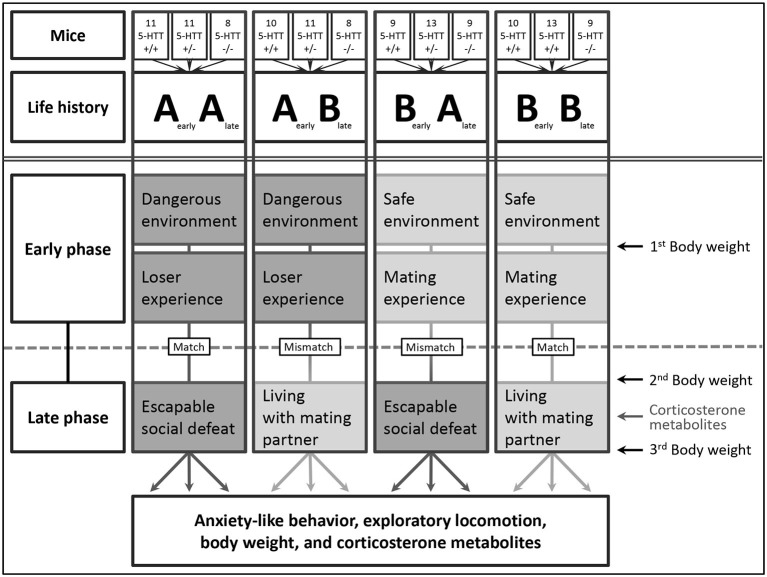
**Overview of the four different life histories**. The figure shows the twelve different experimental groups consisting of male 5-HTT +/+, +/−, and −/− mice, subdivided into four different life histories (AA, AB, BA, and BB). Every life history comprises an early and a late phase, each being either adverse or beneficial. Body weights were measured thrice, corticosterone metabolites were investigated once. Numbers indicate the quantity of animals in each group.

In this study, the impact of either consistent or changing social life experiences on behavioral and hormonal parameters as well as body weight in adult male 5-HTT +/+, +/−, and −/− mice was investigated. For this purpose, four different life histories were experimentally induced in mice of all three 5-HTT genotypes. Each life history consisted of an early and a late phase of either adverse (“A”) or beneficial (“B”) nature. The early phase comprised the prenatal and suckling period until weaning as well as adolescence, the phase during which mice reach sexual maturity. The subsequent phase of early adulthood was here referred to as late phase. While two groups grew up under adverse conditions (“A”), two other groups experienced beneficial conditions during the early phase. In the late phase, mice were exposed to a condition that either matched or mismatched their early phase conditions, when they were either exposed to an escapable adverse situation (“A”) or a beneficial situation (“B”), resulting in four experimental groups (see Figure 1): AA (matched adversity, *n* = 30 (except for 3rd body weight: *n* = 28)), AB (mismatch, *n* = 29), BA (mismatch, *n* = 31), and BB (matched benefit, n = 32). In the following, each experimental phase is described in detail.

#### Breeding

For breeding of the experimental animals, adult naïve 5-HTT +/− females were housed singly 1 day prior to mating (experimental day 0) and received a small amount of soiled bedding originating from the prospective mating partner to stimulate estrus. The next day, an adult sexually experienced 5-HTT +/− male was introduced into the female’s cage and mating was allowed to take place for five consecutive days (experimental days 1–5). Subsequently, each female was housed individually and, in the event of fertilization, gave birth on experimental days 20–24. The day of birth was considered as postnatal day (PND) 1.

#### Early phase: prenatal and suckling period as well as adolescence

In the course of the early phase, comprising the prenatal and suckling period as well as adolescence, experimental males experienced either an adverse (**A**A and **A**B) or beneficial (**B**A and **B**B) environment (Figure 1).

##### Prenatal and suckling period

During pregnancy and lactation, a dangerous or safe environment was created in accordance to the paradigm established by Heiming et al. (2009, 2011). By repeatedly exposing mothers of the **A**A and **A**B groups to soiled bedding from unfamiliar males (unfamiliar male bedding, UMB), the threat of infant killing, and thus an adverse environment, was simulated (vom Saal and Howard, 1982; Elwood and Kennedy, 1991; Figure 1). The unfamiliar males (*n* = 34) were sexually naïve adults from either the NMRI, CD-1, C57Bl/6J, or BALB/c strain. To maximize the “impression of a dangerous environment”, bedding material of several different males was used for the UMB treatment. In contrast, mothers of the **B**A and **B**B groups were treated with fresh sawdust (neutral bedding, NB), which does not signal any specific danger and is thus referred to as safe environment (Figure 1). For each treatment, approximately 220 ml of the respective bedding material was introduced into the cage of the dam in the corner opposite of the nest and left there until the next cage change. Cages were cleaned once a week, except during the late prenatal and early postnatal phases (experimental days 15–27). The treatment took place 5 times during the prenatal phase (experimental days 9, 12, 13, 15, and 16) and 9 times during the postnatal phase until weaning (PND 3–5, 4–7, 6–8, 7–10, 10–12, 11–14, 13–15, 14–17, and 17–19). When aged 22 days, the offspring were separated from the mother and housed in same-sex but mixed-genotype groups of two to five animals. The further experiment was exclusively conducted with the male offspring. In order to enable genotyping of the mice and for the purpose of individual identification, ear cuts were made on the day of weaning.

##### Adolescence

From PND 35 ± 2 on, the males were single housed. Starting at an age of 37 ± 2 days, the male UMB treated offspring (**A**A and **A**B) were provided with mildly adverse social experiences, whereas the male NB treated offspring (**B**A and **B**B) experienced beneficial social situations (Figure 1). In detail, **A**A and **A**B mice were repeatedly confronted with an adult male of the aggressive NMRI strain (Navarro, 1997) in order to provide loser experiences. For this purpose, the experimental male was placed in the home cage (Makrolon type III) of the NMRI male and the behavior was evaluated by an experienced observer, who terminated the confrontations if fighting escalated. To confirm a loser experience, the classification was done according to Jansen et al. (2010). In order to provide the male offspring of the NB condition (**B**A and **B**B) with a beneficial experience, they were exposed to an adult sexually experienced female 5-HTT +/+, +/−, or −/− mouse in estrus. Females of all three 5-HTT genotypes were used in a way ensuring that each male encountered females of at least two genotypes. For the determination of the estrus cycle phases (proestrus, estrus, metestrus, diestrus) of the females, some vaginal smear was taken and analyzed by light microscopy according to the characterization of Allen (1922) at least 15 min before each confrontation. Encounters with a female were conducted in a neutral cage (Makrolon type III) filled with fresh sawdust. Altogether, each experimental subject experienced five encounters with either a male of the NMRI strain or a female of the 5-HTT +/+, 5-HTT +/−, and 5-HTT −/− genotype, respectively. Both adverse and beneficial encounters took place once per day between 9:00 and 10:00 am over a time period of 24 days with an interval of 6 days each (PND 37 ± 2, 43 ± 2, 49 ± 2, 55 ± 2, and 61 ± 2).

#### Late phase: adulthood

At the age of 70 ± 2 days, males were transferred to a custom-made cage system (Lewejohann et al., 2010), where they experienced either an escapable adverse (A**A** and B**A**) or a beneficial social situation (A**B** and B**B**; Figure 1). The cage system consisted of a main cage (Makrolon type III) and smaller “safe” cage (Makrolon type II, 22 × 16 × 14 cm^3^), connected via a water basin (Makrolon type II). The main cage as well as the smaller safe cage was equipped with fresh sawdust, a paper towel, and a metal lid providing food and water *ad libitum*. The water basin was filled with tap water to a height of approximately 3 cm. This cage system served to provide an opportunity to escape from potential agonistic encounters in the main cage through the water basin into the smaller safe cage, offering protection from further attacks. After an acclimatization phase of 24 h, in which the experimental male was individually housed in the cage system with the water basin being empty, the set-up was cleaned and either a dominant male from the aggressive NMRI strain (applied to A**A** and B**A** mice, providing an escapable adverse experience) or a female in estrus (applied to A**B** and B**B** mice, providing a beneficial experience) was placed into the clean main cage. Subsequently, the experimental males were introduced into the main cage (PND 71 ± 2). As part of the encounter of A**A** and B**A** males with an NMRI male, the experimental males were attacked, escaped into the safe cage, and stayed there until they were taken out and placed back into the main cage on the next day so as to escape once again. At the same time, A**B** and B**B** males were allowed to permanently live with a female in the main cage without the need to escape. On 4 out of the following 6 days (PND 72 ± 2, 75 ± 2, 76 ± 2, 77 ± 2) between 8:00 am and 11:00 am, the experimental animals were either placed back into the main cage where the aggressive NMRI males resided (A**A** and B**A**) or shortly handled and returned to the female (A**B** and B**B**). Consequently, the male experimental animals experienced either escapable adversity (A**A** and B**A**) or they had permanent access to a mating partner (A**B** and B**B**) during this time. Escape latencies of A**A** and B**A** mice were neither significantly influenced by genotype (RM ANOVA, Greenhouse-Geisser correction: *F*_(2,55)_ = 2.443, *p* = 0.096), nor previous experiences (A**A** vs. B**A**: *F*_(1,55)_ = 0.236, *p* = 0.629). Since A**B** and B**B** mice did not escape from the main cage, escape latencies were not assessed. In the course of the late phase all male experimental animals were tested for their anxiety-like and exploratory behavior in three standard behavioral tests. During the whole experiment the experimenter was blind to the genotypes.

### Anxiety-like behavior and exploratory locomotion

Behavioral testing took place between PND 75 ± 2 and 77 ± 2 days. In total, 122 males (*n*_AA_ = 30, *n*_AB_ = 29, *n*_BA_ = 31, and *n*_BB_ = 32) were investigated for their anxiety-like and exploratory behavior in the Elevated plus maze test (EPM), the Dark-light test (DL), and the Open field test (OF). The order of tests was the same for each animal with one test per day and followed previous recommendations ranking the tests from least stressful to most stressful (van Gaalen and Steckler, 2000; McIlwain et al., 2001). All behavioral tests were performed during the light phase in a room different from the housing room. The test equipment was thoroughly cleaned with 70% ethanol and dried between subjects. The animal’s movements were recorded by a webcam (Logitech Webcam Pro 9000) and analyzed by the video tracking system ANY-maze (Version 4.75, Stoelting Co., Wood Dale, USA).

#### Elevated plus maze test

At the age of 75 ± 2 days, the mice were tested in the EPM (Pellow et al., 1985; Lister, 1987, 1990). The plus-shaped apparatus consisted of two opposing open arms (30 × 5 cm^2^) and two opposing closed arms (30 × 5 cm^2^) with 20 cm high walls that extended from a central square (5 × 5 cm^2^). The two open arms were enclosed by a small lip (4 mm) preventing the mice from falling off. The plus maze apparatus was elevated 50 cm above the ground and illuminated by a light bulb (150 lx). After spending 1 min in an empty cage, each mouse was individually placed on the central platform facing a closed arm and allowed to freely explore the apparatus for 5 min. The parameters measured were the percentage of time spent on the open arms and the percentage of entries into the open arms to assess anxiety-like behavior, and the sum of entries into the open and closed arms as an indicator of exploratory locomotion.

#### Dark-light test

The DL (Crawley and Goodwin, 1980) was performed at the age of 76 ± 2 days by means of a modified Makrolon cage type III, which was separated into two compartments by a partition including a sliding door. The dark compartment (17 × 27 × 16 cm^3^) was painted black, had an opaque lid, and was unlit, whereas the light compartment (28 × 27 × 16 cm^3^) had transparent walls, no lid, and was illuminated by overhead lighting (570 lx). Each mouse was placed inside the dark compartment with the lid and sliding door closed and remained there for 1 min before the sliding door was opened and the mouse could freely explore the DL apparatus for 5 min. The parameters analyzed were the latency to enter the light compartment and the time spent in the light compartment as indicators of anxiety-like behavior, and the number of entries into the light compartment to assess exploratory locomotion.

#### Open field test

The mice were tested in the OF (Archer, 1973; Treit and Fundytus, 1988) at the age of 77 ± 2 days. The OF consisted of a white square arena (80 × 80 × 42 cm^3^), illuminated by an overhead bulb (600 lx). Each mouse was placed individually inside a cylinder (11 cm diameter, 20 cm high) standing in one corner of the OF apparatus. After 1 min the cylinder was lifted and the mouse was allowed to freely explore the arena for 5 min. The parameters analyzed were the distance the mice traveled for assessing exploratory locomotion and the time they spent in the center of the arena (defined as the area of the OF being located at least 20 cm distant from the walls) to measure anxiety-like behavior.

### Body weights and corticosterone metabolites

#### Body weights

Each experimental male was weighed during the early phase on PND 22, right at the beginning of the late phase on PND 70 ± 2, and at the end of the late phase on PND 77 ± 2 (Figure 1).

#### Corticosterone metabolites

The stress status of the experimental mice during the late phase (PND 75 ± 2, Figure 1) was monitored non-invasively by measuring concentrations of corticosterone metabolites (CM) in the feces (Touma et al., 2003, 2004; Lepschy et al., 2010). Since Touma et al. (2003) showed that a peak of CM can be found in the feces 8–12 h after the exposure to a stressor, all fecal samples were collected between 4:00 pm and 8:00 pm (that is 8–12 h after being either placed back to the NMRI male or handled). For the sample collection, mice were placed individually in standard Makrolon cages type II equipped with three paper towels and food and water provided *ad libitum*. All feces defecated during the 4 h sampling period were collected and frozen at −20°C. The fecal samples were dried and homogenized, and aliquots of 0.05 g were extracted with 1 ml of 80% methanol (Palme et al., 2013). Subsequently, the samples were analyzed by means of a 5α-pregnane-3β,11β,21-triol-20-one enzyme immunoassay, previously established and successfully validated to measure CM in mice (for details see Touma et al., 2003, 2004). Intra- and inter-assay coefficients of variation were below 10% and 12%, respectively.

For validation purposes, an additional fecal sampling was performed 2 days before the late phase (PND 68 ± 2). Using this sample as a reference value the change in stress levels from the first to the second sampling point was analyzed in order to evaluate the glucocorticoid response to the escapable adversity or cohabitation with a female, respectively. The data clearly proved a significant variation in the change in CMs (Univariate ANOVA, *F*_(3,118)_ = 14.653, *p* < 0.001). *Post hoc* analysis revealed a distinctly stronger increase in CM concentrations in mice experiencing escapable adversity in the late phase (mean (ng/0.05 g feces) ± standard error of the mean (SEM): A**A**: 52.6 ± 7.3; and B**A**: 50.5 ± 7.2) compared to mice that cohabitated with a female (A**B**: 4.2 ± 7.4; and B**B**: −2.4 ± 7.1) during this time (Bonferroni, AA vs. AB: *p* < 0.001; AA vs. BB: *p* < 0.001, BA vs. AB: *p* < 0.001; BA vs. BB: *p* < 0.001).

### Statistical analysis

Additional to observed elementary data, the total area under the weight curve (AUC) was calculated with the trapezoidal rule using the time of the three sample points (days) on the abscissa and body weight (g) on the ordinate.

Data were analyzed using General Linear Models (GLM). To meet the assumptions of parametric analysis, residuals were graphically examined for homoscedasticity and outliers and the Lilliefors corrected Kolmogorov-Smirnov Test was applied. When necessary, raw data were transformed using square-root- or logarithmic transformations. Specifically, the following kinds of models were established and fitted to the different dependent variables:

- Model (A) Repeated measures ANOVA (RM ANOVA) of body weight with within-subjects factor “time” (postnatal day, PND), fixed between-subject factors “life history” and “genotype”, and the interaction of “life history” and “time”,
- Model (B) Univariate ANOVA of several dependent variables (anxiety-like and exploratory behaviors, corticosterone meta-bolites) with fixed between-subject factors “life history”, “genotype”, and the interaction of “life history” and “genotype”,
- Model (C) Univariate ANOVA of several dependent variables (anxiety-like and exploratory behaviors) fixed between-subject factors “early phase”, “late phase”, and the interaction of “early phase” and “late phase”.

All main effects and interaction terms were tested on local significance level alpha = 0.05, respectively. If there were significant main or interaction effects, *post hoc* pairwise comparisons of different levels were conducted using Bonferroni adjustment. Data are presented as bars or dots with means and standard error.

All statistical analyses were conducted using the statistical software IBM SPSS Statistics (IBM Version 21, Release 2012). Graphs were created using the software SigmaPlot 12.0 for Windows (Build 12.0.0.182, Systat Software, Inc. 2011).

## Results

### Effects of genotype and life history

The analysis of the data revealed several main effects of both life history and genotype, but no interaction between life history and genotype. In the following the main effects of life history and genotype are described in detail.

#### Body weight and corticosterone metabolites

**Figure 2:**
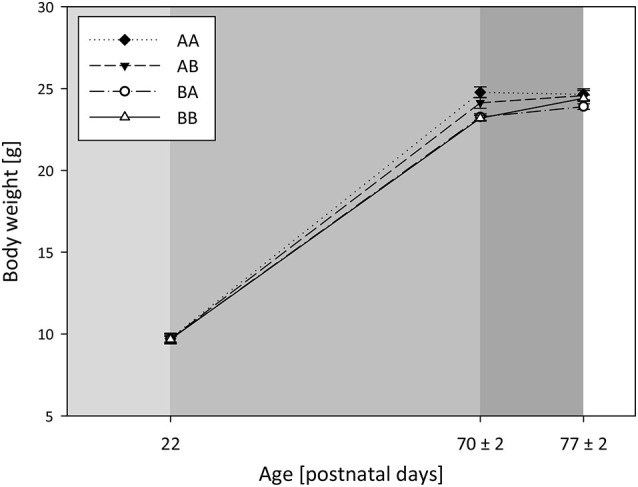
**Body weights: Effects of life history**. Body weights were assessed at weaning (PND 22) after the simulation of either a dangerous or safe environment (light gray), at the end of the early phase (PND 70 ± 2) after 5 loser or mating experiences (gray), and at the end of the late phase (PND 77 ± 2) after escapable defeat or cohabitation with a female (dark gray). Sample sizes: *n*_AA_ = 28, *n*_AB_ = 29, *n*_BA_ = 31, *n*_BB_ = 32). Data are presented as means ± SEM.

Body weights were measured on PND 22, 70 ± 2, and 77 ± 2, and were found to change over the course of life, demonstrated by a main effect of time (Model A, RM ANOVA, Greenhouse-Geisser correction: *F*_(1.5,157.6)_ = 10015.774, *p* < 0.001, Figure 2). *Post hoc* analysis showed that weight increased from PND 22 to 70 ± 2 (Bonferroni, *p* < 0.001) as well as from PND 70 ± 2 to 77 ± 2 (*p* < 0.001). Corticosterone metabolites (CM) were assessed on PND 75 ± 2.

##### Effects of genotype

There was neither a significant main effect of genotype on body weights measured over the three time points (Model A, RM ANOVA, *F*_(2,108)_ = 2.487, *p* = 0.088), nor concerning the area under the weight curve (Univariate ANOVA, *F*_(2,108)_ = 0.485, *p* = 0.617). Likewise, there was no significant effect of genotype on CM in adulthood (Model B, Univariate ANOVA, *F*_(2,110)_ = 1.701, *p* = 0.187; Table 1).

**Table 1:** **Effects of life history and genotype on anxiety-like behavior, exploratory locomotion, and corticosterone metabolites**.

Parameter	Life history				Genotype			ANOVA						Transf.
	AA	AB	BA	BB	+/+	+/−	−/−	Life history		Genotype		Life history × Genotype	
	Mean ± SEM	Mean ± SEM	Mean ± SEM	Mean ± SEM	Mean ± SEM	Mean ± SEM	Mean ± SEM	F	Sig.	F	Sig.	F	Sig.	
**Elevated plus maze test**
Time on open arms (%)	12.2 ± 2.4	13.8 ± 2.3	24.4 ± 3.6	14.0 ± 1.8	20.6 ± 2.4	18.3 ± 2.2	7.9 ± 1.9	5.427	**0.002**	14.408	**0.001**	0.577	0.748	Ang.
Entries into open arms (%)	25.2 ± 2.7	32.3 ± 2.4	35.5 ± 3.0	30.4 ± 2.5	35.7 ± 2.0	33.8 ± 2.0	21.1 ± 2.7	3.387	**0.021**	12.453	**0.001**	0.371	0.896	NT
Sum of entries (#)	22.7 ± 1.3	20.0 ± 1.3	25.1 ± 1.3	21.1 ± 1.1	24.0 ± 0.9	22.6 ± 1.0	19.8 ± 1.4	3.669	**0.015**	4.047	**0.020**	1.402	0.220	NT
**Dark-light test**
Entries into light (#)	4.0 ± 0.7	3.4 ± 0.6	6.5 ± 0.6	4.7 ± 0.6	5.5 ± 0.6	5.5 ± 0.5	2.5 ± 0.5	5.931	**0.001**	13.044	**0.001**	0.639	0.699	Log.
Time in light (s)	28.9 ± 6.2	24.2 ± 5.7	65.7 ± 9.0	53.8 ± 12.4	62.5 ± 10.5	46.6 ± 6.0	17.2 ± 5.1	7.366	**0.001**	13.882	**0.001**	0.826	0.552	SqR.
Latency to light (s)	151.8 ± 23.5	145.5 ± 20.5	67.5 ± 16.7	94.5 ± 18.9	86.0 ± 16.8	89.7 ± 14.3	180.7 ± 20.5	5.175	**0.002**	9.405	**0.001**	0.243	0.961	Log.
**Open field test**
Distance (m)	24.7 ± 1.4	21.8 ± 1.3	29.9 ± 1.0	24.4 ± 1.1	26.4 ± 1.0	26.5 ± 0.9	22.1 ± 1.5	10.129	**0.001**	6.809	**0.002**	1.746	0.117	NT
Time in center (s)	8.0 ± 1.5	7.5 ± 1.3	9.8 ± 1.2	9.5 ± 1.1	10.5 ± 1.1	10.1 ± 1.1	4.6 ± 0.8	2.183	0.094	12.929	**0.001**	1.544	0.171	Ang.
**Corticosterone metabolites**
PND 75 ± 2 (ng/0.05 g)	84.2 ± 11.5	40.2 ± 2.5	91.3 ± 9.2	38.1 ± 2.3	70.3 ± 9.2	54.8 ± 5.0	67.6 ± 8.6	18.714	**0.001**	1.701	0.187	1.175	0.325	SqR.

*Data analysis was performed using general linear models. Data are given as untransformed means of experimental groups ± standard error of the mean (SEM). The statistical analysis (Model B, Univariate ANOVA) is summarized with respect to the transformation used (NT, not transformed; SqR, square root transformation; Log, logarithmic transformation; Ang, angular transformation) and the effects of life history, genotype, and life history-by-genotype-interaction*.

##### Effects of life history

A significant interaction effect of life history and time on body weights over the lifespan could be detected (Model A, RM ANOVA, Greenhouse-Geisser correction: *F*_(4.4,157.6)_ = 5.738, *p* < 0.001), with weights increasing from PND 22 to PND 70 ± 2 in mice of each life history group (Figure 2). Unlike, from PND 70 ± 2 to 77 ± 2 weights did not significantly change in AA mice (*p* = 0.501), whereas they increased in AB (*p* = 0.007), BA (*p* = 0.003), and BB mice (*p* < 0.001)). Moreover, there was a significant main effect of life history on the area under the weight curve (*F*_(3,108)_ = 5.820, *p* = 0.001). *Post hoc* analysis revealed that AA mice gained more weight from the first to the last weighing point compared to both BA (Bonferroni, *p* = 0.004) and BB mice (*p* = 0.004). Furthermore, life history significantly influenced CM concentration in adulthood (Model B, Univariate ANOVA, *F*_(3,110)_ = 18.714, *p* < 0.001; Table 1). As proven by *post hoc* testing, mice that experienced later-life adversity (mean (ng/0.05 g feces) ± SEM: A**A**: 84.2 ± 7.6, and B**A**: 91.3 ± 7.5) had significantly higher CM concentrations than mice experiencing later-life benefits (A**B**: 40.2 ± 7.7 and B**B**: 38.1 ± 7.3; Bonferroni, for all comparisons AA vs. AB, AA vs. BB, BA vs. AB, BA vs. BB: *p* < 0.001).

#### Anxiety-like behavior and exploratory locomotion

Anxiety-like behavior and exploratory locomotion were investigated using the EPM, the DL, and the OF test, which measure the aversion of mice to open and/or brightly illuminated areas.

##### Effects of genotype

**Figure 3:**
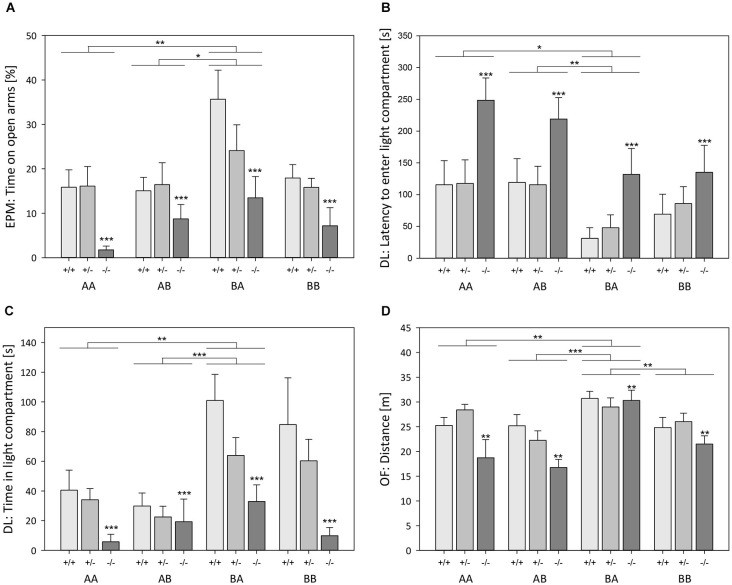
**Behavioral tests: Effects of life history and genotype. (A)** Time spent on open arms of Elevated plus maze test (EPM), **(B)** latency to enter light compartment of Dark-light test (DL), **(C)** time spent in light compartment of DL, and **(D)** distance traveled in Open field test (OF), displayed by male 5-HTT +/+, 5-HTT +/−, and 5-HTT −/− mice grown up in an early adverse (AA and AB) or beneficial (BA and BB) environment and provided with later matching (AA and BB) or mismatching (AB and BA) living conditions in adulthood. Data are given as means ± SEM. Statistics: ANOVA; *post hoc* testing: Bonferroni. *p ≤ 0.05; **p ≤ 0.01; ***p ≤ 0.001. Asterisks above 5-HTT −/− bars indicate differences between 5-HTT −/− and both 5-HTT +/− and 5-HTT +/+ mice. Sample sizes: *n*_AA_ = 30 (11 +/+, 11 +/−, 8 −/−), *n*_AB_ = 29 (10 +/+, 11 +/−, 8 −/−), *n*_BA_ = 31 (9 +/+, 13 +/−, 9 −/−), *n*_BB_ = 32 (10 +/+, 13 +/−, 9 −/−).

ANOVA detected a significant main effect of genotype on the anxiety-like behavior (Table 1) as assessed by the relative time spent on open arms (Model B, Univariate ANOVA, *F*_(2,110)_ = 14.408, *p* < 0.001; Figure 3A) and the relative entries into open arms of the EPM (*F*_(2,110)_ = 12.453, *p* < 0.001). There was also a main effect on the latency to enter the light compartment (*F*_(2,110)_ = 9.405, *p* < 0.001; Figure 3B) and the time spent in the light compartment of the DL (*F*_(2,110)_ = 13.882, *p* < 0.001; Figure 3C) as well as on the time in the center of the OF (*F*_(2,110)_ = 12.929, *p* < 0.001). *Post hoc* analysis revealed that 5-HTT −/− mice spent less time on the open arms of the EPM, made relatively fewer entries into the open arms of the EPM, entered the light compartment of the DL later, and spent less time in the light compartment of the DL as well as in the center of the OF compared to both 5-HTT +/+ and 5-HTT +/− mice (Bonferroni, for all comparisons *p* < 0.001, except for the latency to enter the light compartment of DL: 5-HTT −/− vs. +/−: *p* = 0.002).

The locomotor activity in the test apparatuses was also significantly influenced by genotype (Table 1), which was reflected by the sum of entries into open and closed arms of the EPM (*F*_(2,110)_ = 4.047, *p* = 0.020), the number of entries into the light compartment of the DL (*F*_(2,110)_ = 13.044, *p* < 0.001), and the distance traveled in the OF (*F*_(2,110)_ = 6.809, *p* = 0.002; Figure 3D). Further analysis demonstrated that 5-HTT −/− mice exhibited fewer entries into open and closed arms than 5-HTT +/+ mice (Bonferroni, *p* = 0.025). Moreover, 5-HTT −/− mice entered the light compartment less often than 5-HTT +/+ and +/− mice (for both comparisons *p* < 0.001) and covered significantly shorter distances in the OF compared to both, 5-HTT +/+ (*p* = 0.011) and 5-HTT +/− mice (*p* = 0.005).

##### Effects of life history

Anxiety-like behavior was distinctly influenced by life history (Table 1), as measured by the relative time spent on open arms (Model B, Univariate ANOVA, *F*_(3,110)_ = 5.427, *p* = 0.002; Figure 3A), relative entries into open arms (*F*_(3,110)_ = 3.387, *p* = 0.021), latency to enter the light compartment (*F*_(3,110)_ = 5.175, *p* = 0.002; Figure 3B), and the time spent in the light compartment (*F*_(3,110)_ = 7.366, *p* < 0.001; Figure 3C). *Post hoc* analysis revealed that BA mice spent relatively more time on the open arms (Bonferroni, *p* = 0.003), made relatively more entries into the open arms (*p* = 0.025), entered the light compartment faster (*p* = 0.039), and stayed there longer (*p* = 0.002) than AA mice. Additionally, BA mice differed from AB mice in terms of increased time on the open arms (*p* = 0.044) and in the light compartment (*p* = 0.001), as well as shorter latencies to enter the light compartment (*p* = 0.003).

Life history was also found to have an effect on locomotor activity (Table 1), demonstrated by the sum of entries into open and closed arms (*F*_(3,110)_ = 3.669, *p* = 0.015), the number of entries into the light compartment (*F*_(3,110)_ = 5.931, *p* = 0.001), and the distance traveled in the OF (*F*_(3,110)_ = 10.129, *p* < 0.001; Figure 3D). Further analysis showed that the sum of entries into open and closed arms was increased in BA mice in comparison with AB mice (Bonferroni, *p* = 0.020). BA mice also exhibited more entries into the light compartment than AA (*p* = 0.006) and AB mice (*p* = 0.003). Likewise, the distance traveled in the OF was significantly longer in BA animals compared to every other life history group (BA vs. AA: *p* = 0.008, BA vs. AB: *p* < 0.001, BA vs. BB: *p* = 0.004).

### Effects of early vs. late phase on anxiety-like behavior and exploratory locomotion

Living conditions were the same for AA and AB mice and for BA and BB mice during early phases and for AA and BA mice as well as AB and BB animals during adulthood, respectively. To investigate specific effects of early vs. late phases on anxiety-like behavior and exploratory locomotion and to understand which phases shape individual life histories how, a further analysis was conducted including the early phase and late phase as fixed factors.

Due to the absence of a significant interaction effect between genotype and life history on anxiety-like and exploratory behavior in the preceding analysis (see Table 1), further analyses were conducted without genotype as between-subject factor. The analysis revealed significant main effects of the early and of the late phase as well as interactions between the two factors.

#### Effects of early phase

**Figure 4:**
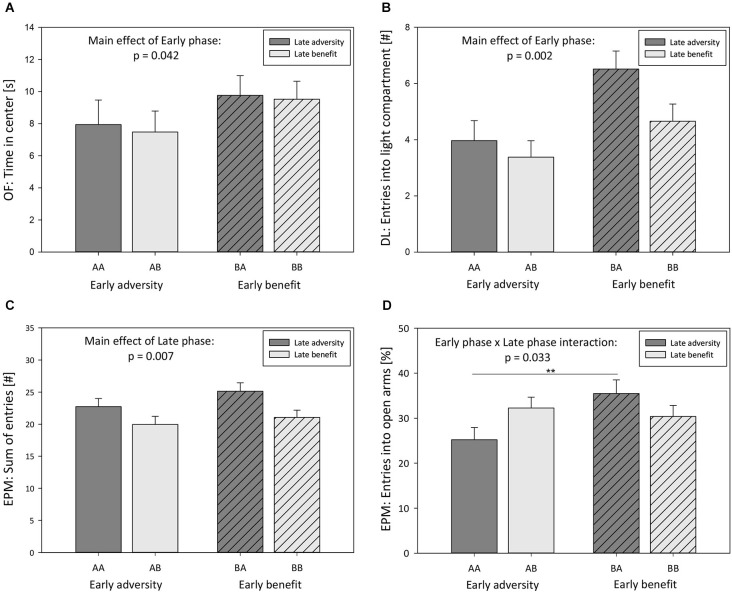
**Behavioral tests: Effects of early and late phase of life. (A)** Time in center of Open field (OF), **(B)** entries into light compartment of Dark-light test (DL), **(C)** sum of entries into open and closed arms of Elevated plus maze test (EPM), and **(D)** entries into open arms of EPM displayed by male 5-HTT +/+, 5-HTT +/−, and 5-HTT −/− mice grown up in an early adverse (AA and AB) or beneficial (BA and BB) environment and provided with matching adverse (AA), matching beneficial (BB) or mismatching (AB and BA) living conditions in adulthood. Data are given as means ± SEM. Statistics: ANOVA; *post hoc* testing: Bonferroni. Sample sizes: *n*_early adversity_ = 59, *n*_early benefit_ = 63, *n*_late adversity_ = 61, *n*_late benefit_ = 61.

Experiences during early phases of life were found to significantly influence anxiety-like behavior in adulthood (Model C, Univariate ANOVA, EPM: relative time spent on open arms: *F*_(1,118)_ = 6.103, *p* = 0.015; DL: latency to enter light compartment: *F*_(1,118)_ = 10.886, *p* = 0.001; DL: time in light compartment: *F*_(1,118)_ = 14.985, *p* < 0.001; OF: time in center: *F*_(1,118)_ = 4.235, *p* = 0.042; Figure 4A; Table 2). Furthermore, there was a main effect of the early phase on exploratory locomotion (DL: entries into light compartment: *F*_(1,118)_ = 9.897, *p* = 0.002; Figure 4B; OF: distance traveled: *F*_(1,118)_ = 10.746, *p* = 0.001; Table 2). The experience of an adverse early environment was associated with increased anxiety-like behavior and decreased exploratory locomotion in comparison with beneficial early conditions.

**Table 2:** **Effects of early vs. late phase on anxiety-like behavior and exploratory locomotion**.

Parameter	Early phase		Late phase		ANOVA						Transf.
	Early adversity	Early benefit	Late adversity	Late benefit	Early phase		Late phase		Early × late phase	
	Mean ± SEM	Mean ± SEM	Mean ± SEM	Mean ± SEM	F	Sig.	F	Sig.	F	Sig.
**Elevated plus maze test**
Time on open arms (%)	13.0 ± 1.7	19.1 ± 2.1	18.4 ± 2.3	13.9 ± 1.4	6.103	**0.015**	1.168	0.282	4.645	**0.033**	Ang.
Entries into open	28.7 ± 1.9	32.9 ± 2.0	30.4 ± 2.1	31.3 ± 1.7	2.439	0.121	0.134	0.715	5.152	**0.025**	NT
arms (%)
Sum of entries (#)	21.4 ± 0.9	23.1 ± 0.9	24.0 ± 0.9	20.5 ± 0.8	1.950	0.165	7.468	**0.007**	0.270	0.605	NT
**Dark-light test**
Entries into light (#)	3.7 ± 0.5	5.6 ± 0.5	5.3 ± 0.5	4.0 ± 0.4	9.897	**0.002**	2.258	0.136	1.484	0.226	Log.
Time in light (s)	26.6 ± 4.2	59.6 ± 7.7	47.6 ± 5.9	39.7 ± 7.2	14.985	**0.001**	1.798	0.183	0.935	0.336	SqR.
Latency to light (s)	148.7 ± 15.5	81.2 ± 12.6	109.0 ± 15.2	118.7 ± 14.2	10.886	**0.001**	2.315	0.131	0.187	0.666	Log.
**Open field test**
Distance (m)	23.2 ± 1.0	27.1 ± 0.8	27.3 ± 0.9	23.1 ± 0.8	10.746	**0.001**	12.403	**0.001**	1.175	0.281	NT
Time in center (s)	7.7 ± 1.0	9.6 ± 0.8	8.9 ± 1.0	8.6 ± 0.9	4.235	**0.042**	0.025	0.876	0.001	0.998	Ang.

*Data analysis was performed using general linear models. Data are given as untransformed means of experimental groups ± standard error of the mean (SEM). The statistical analysis (Model C, Univariate ANOVA) is summarized with respect to the transformation used (NT, not transformed; SqR, square root transformation; Log, logarithmic transformation; Ang, angular transformation) and the effects of early phase, late phase, and early-by-late phase-interaction*.

#### Effects of late phase

Effects of late phase environmental conditions on the behavior in adulthood was exclusively observed in parameters assessing exploratory locomotion (Model C, Univariate ANOVA, EPM: sum of entries into open and closed arms: *F*_(1,118)_ = 7.468, *p* = 0.007; Figure 4C; OF: distance traveled: *F*_(1,118)_ = 12.403, *p* = 0.001; Table 2). Escapable adversity in adulthood was linked to a markedly increased sum of entries into open and closed arms of the EPM as well as to a longer distance traveled in the OF. Anxiety-like behavior was not affected by late phase environmental conditions.

#### Interaction effects of early and late phase

Additionally, a significant early phase-by-late phase interaction was detected concerning the relative time spent on open arms (Model C, Univariate ANOVA, *F*_(1,118)_ = 4.645, *p* = 0.033) as well as the relative entries into open arms of the EPM (*F*_(1,118)_ = 5.152, *p* = 0.025; Figure 4D; Table 2). *Post hoc* testing revealed that early beneficial conditions caused lower levels of anxiety-like behavior in mice experiencing later adverse conditions (BA) compared to later beneficial conditions (BB, Bonferroni, relative time spent on open arms, *p* = 0.022). Furthermore, later-life escapable adversity led to higher levels of anxiety-like behavior in mice that made prior adverse experiences during early life (AA) compared to mice that experienced early beneficial conditions (BA; relative time spent on open arms: *p* = 0.001; relative entries into open arms: *p* = 0.008; Figure 4D).

## Discussion

The present study was designed to investigate the impact of varying 5-HTT expression and life history on anxiety-like and exploratory behavior, body weights, and non-invasively assessed CM from fecal samplings. For this purpose, mice with genetically driven differences in 5-HTT expression were provided with four different experimentally induced life histories ranging from the prenatal phase through adolescence to adulthood. For the simulation of different socio-environmental conditions, exclusively ecologically relevant stimuli were applied.

### Effects of genotype

Significant genotype-dependent differences in the commonly used tests for anxiety-like behavior and exploratory locomotion were found, with 5-HTT −/− mice displaying increased anxiety-like and decreased exploratory behavior compared to both 5-HTT +/− and 5-HTT +/+ animals, which is in line with previous studies (Kalueff et al., 2007; Heiming et al., 2009; Jansen et al., 2010; Araragi and Lesch, 2013). Consequently, the 5-HTT knockout mouse model represents a valuable model system of the behavioral symptoms produced by the low-expressing variant of the 5HTTLPR in humans, enabling the analysis of the effects of reduced or absent 5-HTT on behavior and the underlying neural and molecular mechanisms.

There was no interaction between the 5-HTT genotype and life history, although it is a well supported phenomenon that the 5-HTT genotype can moderate the effects of environmental stimuli on the behavioral profile (e.g., Caspi et al., 2003; Carola et al., 2008). However, there are also several published studies that could not find such interaction effects (e.g., Risch et al., 2009; Kloke et al., 2013). It can furthermore be taken into account that studies in humans suggest females to be more susceptible to anxiety disorders and depression than males (Breslau et al., 1995; Eley et al., 2004). In the present experimental protocol, it remains to be determined if this is also the case.

No influence of genotype was detected on body weights. Therefore, 5-HTT function did apparently not play a major role in modulating growth (but see Holmes et al., 2002). Likewise, CM concentrations—as indicator of stress—were not significantly influenced by an abolished 5-HTT function, whether complete or partial, which is in accordance with findings of Jansen et al. (2010) and Kloke et al. (2011; but also see Lanfumey et al., 2000; Tjurmina et al., 2002).

### Effects of life history—benefits of adversity?!

In accordance with our hypothesis, life history had a profound effect on the behavioral profile in adulthood. Animals that grew up under early beneficial conditions and were confronted with later escapable adversity (BA), exhibited decreased anxiety-like and increased exploratory behavior in comparison with AA and AB and in some cases even with BB mice. Surprisingly, throughout beneficial experiences made by BB mice did neither cause lower levels of anxiety-like behavior nor increased exploratory locomotion compared to the accumulating adversity experienced by AA mice. Yet, it can not be excluded that the conditions designated as beneficial have been experienced slightly differently by the experimental males (for a review on how serotonin affects sexual behavior see Kiser et al., 2012). However, also in human studies, the absence of adversity has been reported to be not inevitably associated with optimal outcomes (Seery et al., 2010, 2013). There even is theoretical and empirical evidence that some adversity can have a positive effect, while this is not the case for either sheltering from all stressors or continuous stress exposure (Dienstbier, 1989; Fontana and Rosenheck, 1998; Parker et al., 2004, 2006; Seery et al., 2010, 2013). Sheltering is assumed to prevent individuals from developing coping strategies, while the experience of some adverse events is suggested to promote the ability to better cope with challenges (Seery et al., 2010). In a multi-year longitudinal study in humans, Seery et al. (2010) found that a history of some lifetime adversity predicts relatively lower global distress, lower self-rated functional impairment, fewer posttraumatic stress symptoms, and higher life satisfaction over time compared with both, a history of severe adversity and no history of adversity. Further evidence from animal studies comes from Parker et al. (2004, 2006), who found that young squirrel monkeys that were exposed to moderate levels of adversity during development displayed greater resilience to subsequent stressors compared to monkeys without any stress exposure. Thus, as already argued by Sachser (2001), a life without challenge does obviously not *per se* imply a good life, which might explain why BB mice did not exhibit lower levels of anxiety than AA and AB mice.

However, a challenge in life may also not overtax the individual’s capacity to cope (Sachser, 2001). This becomes particularly relevant when addressing the question why lower levels of anxiety-like behavior and more exploratory locomotion were found in BA compared to AA mice. Both groups had to face the same situation of escapable adversity during the late phase. Unlike inescapable stress, which is well documented to potentiate anxiety-like behavior, escapable stress does not elicit this effect (Korte et al., 1999; Korte and De Boer, 2003). The behavioral differences between AA and BA mice are thus apparently based on previous experiences and especially on the combination of early and later life events. While the accumulation of adverse experiences during the early phase, followed by escapable adversity in later life (AA), results in relatively high levels of anxiety-like behavior and decreased locomotor activity, the same late escapable adversity causes less anxiety-like and more exploratory behavior when following on beneficial early experiences (BA).

This finding is underlined by our additional early vs. late phase analysis, revealing that anxiety-like behavior is prevalently influenced by early phase environmental experiences, but also by early-by-late phase interactions. While lower levels of anxiety-like behavior can be traced to a beneficial early phase, adverse early conditions obviously triggered increased anxiety-like behavior in adulthood. This result is in line with earlier studies linking adverse early experiences with increased levels of anxiety (Vallée et al., 1997; Meaney, 2001; Pryce et al., 2005; Carola et al., 2008; Heiming et al., 2009). Additionally, interactions exist between early and late phase experiences in modulating anxiety-like behavior. Namely, adverse early life events cause high levels of anxiety when further adversity in the late phase accumulates (AA), whereas late escapable adversity has the opposite effect when following on early beneficial conditions (BA) as shown by distinctly less anxiety-like behavior. Experiences during the late phase, however, do not account for the level of anxiety-like behavior in adulthood. Yet, late phase life events as well as early experiences have a strong impact on exploratory locomotion. The higher exploratory activity in animals experiencing adverse late conditions can thus be attributed to the escapable adversity in the present study, which is consistent with findings of Korte et al. (1999) who found a similar effect in rats exposed to escapable footshock stress. Our findings thus confirm what has been suggested recently: Early experiences shape some aspects of the behavioral profile, such as levels of anxiety, while later experiences enable final adjustments to the current environmental demands (Sachser et al., 2013).

Considering the two competing hypotheses that address the impact of life history on anxiety, the allostatic load and the mismatch hypothesis (see Section Introduction), we would have expected high levels of anxiety either in individuals experiencing accumulating adversity or in individuals that experienced a discrepancy between early and later living conditions. Surprisingly, neither AA mice of the presumed “allostatic load group” nor BA mice of the “mismatch group”, consisting of an early beneficial and a later adverse life, displayed increased anxiety-like behavior compared to AB and BB mice. What we actually found were lower levels of anxiety-like behavior in the BA mismatch group compared to AA and AB mice. Therefore, the results concerning anxiety-like behavior support none of the two hypotheses.

Apart from effects on measures of anxiety and exploration, the present study also revealed an influence of life history on body weights, though not regarding the simulated dangerous (UMB) or safe (NB) environment during the prenatal and suckling period. These conditions did not affect the growth of the pups, as no differences between these groups were detected on the day of weaning (PND 22). In contrast, loser experiences as compared to mating experiences during adolescence had an effect on body weights measured at the end of the early phase (PND 70 ± 2), with five loser experiences (**A**A and **A**B) causing more weight gain than five mating experiences (**B**A and **B**B). These results are comparable with findings of other studies, where the experience of social defeat was also associated with increased body weight (Bartolomucci et al., 2004; Nestler, 2012; but for the opposite effect see Sachser, 1993; Tamashiro et al., 2007). Interestingly, although A**A** and B**A** mice were confronted with the same escapable adversity during the late phase, the effects on body weights (PND 77 ± 2) were completely different: AA mice that experienced accumulating adversity throughout their lives lost weight during the adverse late phase, while this was not the case for BA mice. Notably, stress levels, indicated by CM concentrations, were similarly increased in AA as well as BA mice irrespective of prior living conditions, suggesting the condition to be a meaningful, ecologically relevant stressor for both groups. Furthermore, the increased CM levels in AA mice indicate that social defeat stress, unlike many other environmental stressors, does not result in habituation upon repeated presentation, which confirms previous findings (Tornatzky and Miczek, 1993). As a result, stress during adulthood results in weight loss only when the previous life was already characterized by adversity.

## Conclusion

With our study of life history effects in the 5-HTT knockout mouse model we confirm the major role of 5-HTT in shaping an individual’s behavior, and contribute to an understanding of how behavioral profiles are shaped by quality and timing of socio-environmental experiences during life. Surprisingly, a throughout beneficial life does not necessarily lead to lower levels of anxiety compared to a life characterized by accumulating adversity. Neither do escapable adverse events in later life *per se* cause increased anxiety-like behavior. Rather, as one of the major findings, we provide evidence that some lifetime adversity can have a positive effect. These results stress the need for comprehensive information on an individual’s life history to gain a deeper understanding about how adaptive mechanisms can regulate anxiety. Thus, our findings have important implications for future studies and biomedical research because sheltering from all stressors becomes increasingly controversial as ultimate goal in the prevention of diseases. Since similar findings have been observed in humans and animals, further studies may help to shed light on the benefits of adversity.

## Conflict of interest statement

The authors declare that the research was conducted in the absence of any commercial or financial relationships that could be construed as a potential conflict of interest.
